# Associations of estimated glucose disposal rate and overactive bladder and the potential mediating role of systemic inflammation: NHANES 2005–2018

**DOI:** 10.1186/s13098-026-02182-4

**Published:** 2026-05-19

**Authors:** Xiaodan Wu, Lidi Zhong, Yanrong Chen, Qin Du, Yan Li, Qinghui Liao, Min Liu, Kangbao Lai, Ping Liu

**Affiliations:** Department of Endocrinology and Metabolism, The Second Affiliated Hospital, School of Medicine, The Chinese University of Hong Kong (Shenzhen) & Longgang District People’s Hospital of Shenzhen, No. 53, Aixin Road, Longgang District, Shenzhen, 518172 China

**Keywords:** Inflammation, Estimated glucose disposal rate, Insulin resistance, Overactive bladder, NHANES

## Abstract

**Background:**

Insulin resistance (IR) is linked to overactive bladder (OAB), with chronic inflammation serving as a common pathophysiological mechanism in both conditions. As a robust marker of IR, estimated glucose disposal rate (eGDR) was selected as the exposure variable to examine its relationship with OAB, with a specific focus on the mediating role of inflammation.

**Methods:**

We conducted a cross-sectional study using data from the 2005–2018 National Health and Nutrition Examination Survey (NHANES). We utilized weighted logistic regression to assess the association of eGDR with OAB, while restricted cubic splines (RCS) helped characterize potential nonlinear patterns in this relationship. Subgroup analyses and interaction tests were performed to assess heterogeneity in the eGDR-OAB association across different subgroups. Mediation analysis was used to explore whether systemic inflammatory biomarkers statistically explain part of the eGDR-OAB association, while acknowledging that causal inference is limited by the cross-sectional design.

**Results:**

31,351 participants were included in this study, of whom 6,356 (20.27%) were diagnosed with OAB. In the fully adjusted Model, each one-unit increment in eGDR was associated with a 11% reduction in OAB risk (OR = 0.89; 95% CI: 0.87–0.91; *P* < 0.001) and a 48% decreased risk of OAB for the highest eGDR quartile (Q4) compared to the lowest quartile (Q1) (OR = 0.52; 95% CI: 0.42–0.64; *P* < 0.001). RCS analysis showed a significant nonlinear inverse association between eGDR and OAB, and this association was consistently observed across nearly all subgroups. Mediation analyses revealed that systemic inflammatory biomarkers statistically mediated the relationship between eGDR and OAB, explaining approximately 0.64–3.15% of the total effect, suggesting that inflammation accounts for only a small fraction of the eGDR–OAB association, while other mechanisms (e.g., autonomic dysfunction, microvascular damage, urothelial changes) likely contribute more substantially.

**Conclusions:**

Our research confirmed the correlation between eGDR, inflammation and OAB. The potential mediating effect of inflammation between eGDR and OAB provides a meaningful direction for future research and targeted interventions.

**Supplementary Information:**

The online version contains supplementary material available at 10.1186/s13098-026-02182-4.

## Introduction

Overactive bladder (OAB) is a prevalent chronic urological syndrome. According to the standardized definition provided by the International Continence Society, OAB is characterized primarily by urinary urgency, which may be accompanied by urge urinary incontinence (UUI), and is frequently associated with nocturia and increased daytime frequency, in the absence of a urinary tract infection or other overt pathology [1, 2]. The pooled prevalence of OAB, derived from a meta-analysis of 53 published studies, was estimated at 20%, highlighting its widespread impact [3]. OAB significantly impairs patients’ quality of life and is associated with sleep disturbances, anxiety, and depression [4]. Furthermore, it poses a substantial and growing public health burden [5].

The role of insulin resistance (IR) as a shared pathological basis for metabolic conditions is increasingly recognized, with mounting evidence connecting it to elevated OAB risk [6, 7]. The hyperinsulinemic-euglycaemic clamp represents the gold standard method for evaluating insulin sensitivity, offering the most precise assessment of insulin resistance. However, its invasiveness and complexity restrict its use in routine clinical practice [8]. As a result, validated surrogate indices—including the homeostasis model assessment of insulin resistance (HOMA-IR) and the triglyceride-glucose (TyG) index—have been proposed [9, 10]. The estimated glucose disposal rate (eGDR), a practical and validated index incorporating waist circumference, hypertension status, and glycated hemoglobin (HbA1c), is now widely used in population-based studies to assess IR [11]. Although previous studies have examined the relationship between various indices of IR and OAB [12, 13], the specific association between eGDR and OAB has not yet been clearly established. Moreover, the biological pathways potentially underlying this relationship remain incompletely understood.

Compared with single-marker indices such as TyG or HOMA-IR, eGDR integrates multiple metabolic domains, potentially offering a more comprehensive assessment of IR. Systemic chronic inflammation represents a key pathological link between IR and multiple diseases, including OAB. IR can be driven by several cellular mechanisms, such as oxidative stress, endoplasmic reticulum stress, lipotoxicity, and glucotoxicity, all of which can initiate inflammatory responses or be exacerbated in the presence of existing inflammation [14]. Both persistent systemic inflammation and localized inflammation of the bladder urothelium are believed to be directly implicated in the etiology of OAB [15]. Emerging systemic inflammatory biomarkers, including neutrophil-to-lymphocyte ratio (NLR), monocyte-to-lymphocyte ratio (MLR), neutrophil-monocyte-to-lymphocyte ratio (NMLR), systemic immune-inflammation index (SII), systemic inflammation response index (SIRI), and aggregate index of systemic inflammation (AISI), are increasingly favored in epidemiological and clinical studies. These biomarkers are easily accessible, low-cost, and provide a robust reflection of systemic inflammatory activity [16, 17]. We therefore hypothesize that IR may elevate OAB risk by promoting systemic inflammation.

## Materials and methods

### Study design and sample population

Data for this cross-sectional analysis were sourced from the National Health and Nutrition Examination Survey (NHANES), a recurring, nationally representative survey conducted in two-year cycles. NHANES employs a sophisticated, multistage probability sampling strategy to gather health and nutritional data from a non-institutionalized US population, encompassing both adults and children. The survey protocol was approved by the National Center for Health Statistics Research Ethics Review Board, and all participants provided written informed consent.

**Fig. 1:**
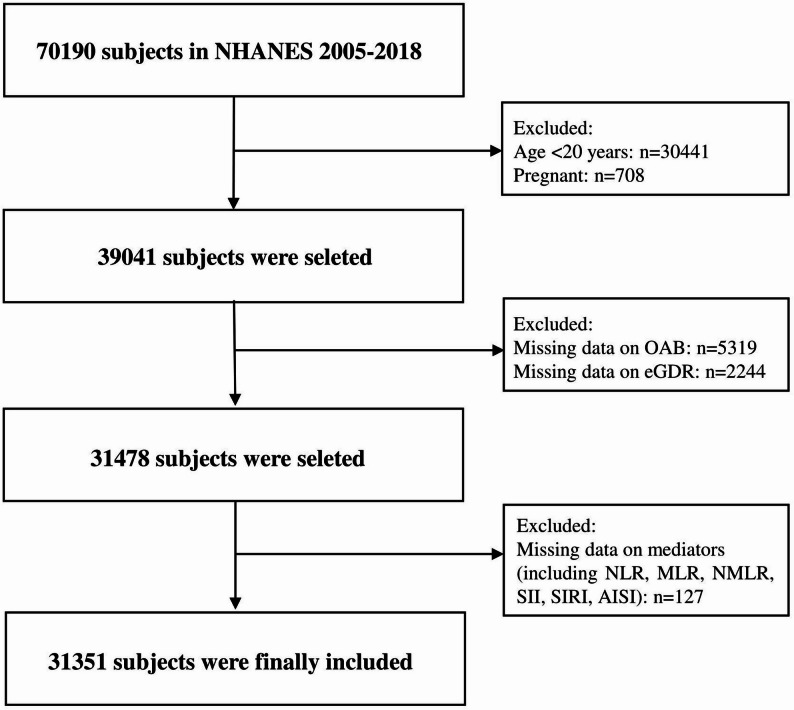
Flowchart of enrollment. NHANES: National Health and Nutrition Examination Survey; OAB, overactive bladder; eGDR, estimated glucose disposal rate; NLR, neutrophil-to-lymphocyte ratio; MLR, monocyte-to-lymphocyte ratio; NMLR, neutrophil-monocyte-to-lymphocyte ratio; SII, systemic immune-inflammation index; SIRI, systemic inflammation response index; AISI, aggregate index of systemic inflammation

This analysis drew on data from seven consecutive NHANES cycles (2005–2018), initially including 70,190 participants. We excluded individuals who were under the age of 20 (*n* = 30,441), pregnant (*n* = 708), missing data on OAB diagnosis (*n* = 5,319), missing eGDR data (*n* = 2,244), or missing data on systemic inflammatory biomarkers, including NLR, MLR, NMLR, SII, SIRI, and AISI (*n* = 127). Following these exclusions, 31,351 participants remained for the final analytic cohort (Fig. 1).

### Assessment of OAB

OAB is clinically characterized by UUI and nocturia [2]. Information was gathered from participants through face-to-face interviews using standardized questionnaires administered by trained staff. The severity of UUI was evaluated using two validated questions regarding occurrence and frequency; nocturia was assessed by querying the average number of nighttime voids over the past 30 days. In this study, OAB was quantified using the Overactive Bladder Symptom Score (OABSS) [18]. Consistent with established diagnostic criteria reported in the literature, a clinical cutoff of ≥ 3 on the OABSS total score was applied to identify OAB cases [12, 13, 19]. Detailed diagnostic criteria for OAB are presented in Table S1.

### Assessment of eGDR 

eGDR was calculated based on the following formula: eGDR = 21.158 - [0.09*waist circumference (cm)] - [3.407* hypertension (yes = 1/no = 0)] - [0.551*HbA1c (%)] [11]. A diagnosis of hypertension was assigned to participants if they met any of the following conditions: systolic blood pressure ≥ 140 mmHg, diastolic blood pressure ≥ 90 mmHg, physician-diagnosed hypertension, or ongoing treatment with antihypertensive drugs. eGDR was categorized into quartiles to facilitate trend analysis, with the lowest quartile (Q1) defined as the reference group against which other quartiles were compared.

### Systemic inflammatory biomarkers

Systemic inflammatory biomarkers included NLR, MLR, NMLR, SII, SIRI, and AISI. The specific formulas and calculation details are presented in Table S2.

### Assessment of other variates

Based on prior literature, we identified potential covariates that may confound the association under study, including age, gender, ethnicity, marital status, education, poverty-to-income ratio (PIR), smoking status, drinking status, physical activity, body mass index (BMI), and hyperlipidemia [13, 20, 21]. Participants were categorised into three groups based on smoking history and current smoking status: (1) never smokers (fewer than 100 cigarettes in lifetime), (2) former smokers (≥ 100 cigarettes ever smoked but currently abstinent), and (3) current smokers (≥ 100 cigarettes ever smoked and active tobacco use). Drinking status was evaluated through self-reported consumption of ≥ 1 standard drink (12 oz beer, 5 oz wine, or 1.5 oz liquor) within the past year, with participants categorized as drinkers or non-drinkers accordingly. Participants were categorised into low and high physical activity based on their self-reported weekly minutes of moderate or vigorous activity. Weight status was defined according to BMI cut-offs: underweight (< 18.5 kg/m²), normal weight (18.5–24.9 kg/m²), overweight (25.0–29.9 kg/m²), and obese (≥ 30.0 kg/m²). Individuals were categorized as having hyperlipidemia if their lipid profile met diagnostic thresholds or if they were currently prescribed lipid-lowering therapy [22]. Participants were classified as having diabetes if they met at least one of the following diagnostic criteria: self-reported physician-diagnosed diabetes, current use of insulin or oral hypoglycemic agents, HbA1c ≥ 6.5%, fasting plasma glucose ≥ 126 mg/dL, or 2-hour postprandial glucose ≥ 200 mg/dL. More details about the covariate data were available at https://www.cdc.gov/nchs/nhanes/.

### Statistical analyses

In accordance with NHANES Analytic Guidelines, we computed sample weights to account for the complex survey design. Specifically, we combined 7 NHANES cycles (2005–2018) and created new weights by dividing the original 2-year MEC weights (WTMEC2YR) by 7, following NHANES analytic guidelines for multi-cycle analyses. All weighted analyses accounted for survey strata (SDMVSTRA) and primary sampling units (SDMVPSU). We performed multiple imputation using chained equations (m = 5 imputations) for variables with > 5% missing data (PIR and alcohol intake). The imputation model included all covariates and the outcome variable. Sensitivity analyses restricted to complete-case data are presented in Table S3. Since none of the continuous variables in this study had a normal distribution, weighted Kruskal-Wallis tests were performed for the comparison of continuous variables, which were expressed as medians and interquartile ranges (IQRs). For the categorical variables, the weighted chi-square test was employed for analysis, with the data presented in the form of weighted percentages. To explore the link between eGDR and OAB, weighted logistic regression models were utilized. In addition to being continuous variables, eGDR was presented as categorical variables grouped in quartiles, with reference to the lowest quartile. This relationship was assessed through three models: Model 1 (unadjusted), Model 2 (adjusted for age, gender and ethnicity), and Model 3 (adjusted for variables in Model 2 and additionally incorporating marital status, education level, PIR, smoking status, drinking status, physical activity, BMI, and hyperlipidemia).

We utilized restricted cubic splines (RCS) models to characterize the potential nonlinear nature of the eGDR-OAB relationship. To evaluate potential bias introduced by imputation for missing covariate data, we performed a sensitivity analysis restricted to participants with complete covariate data. In order to compare whether eGDR is superior to IR indicators such as HOMA-IR and TyG in assessing the risk of OAB, we analyzed the association between HOMA-IR and TyG with OAB using three models in the imputed data. Subgroup analyses were performed across a range of demographic, behavioral, and clinical characteristics, including age, gender, ethnicity, marital status, education, smoking status, drinking status, physical activity, BMI, and the presence of hyperlipidemia, hypertension, or diabetes. Effect modification by these subgroups was tested by introducing interaction terms into the regression models.

We performed mediation analysis to evaluate whether systemic inflammatory biomarkers transmit the link between eGDR and OAB.

All data processing and statistical analyses were conducted in R version 4.4.0, along with Zstats Platform (https://www.zstats.cn/sta/79).

## Results

### Population characteristics

Of the 31,351 participants enrolled, 6,356 (20.27%) were diagnosed with OAB, with the remaining 24,995 (79.73%) serving as the non-OAB group. Compared with non-OAB participants, individuals with OAB were characterized by a cluster of adverse socioeconomic, behavioral, and clinical factors. These included being older, female, Non-Hispanic Black, having a lower PIR, and reporting current or former smoking or alcohol abstinence. This group also demonstrated a higher prevalence of being widowed, divorced, or separated, alongside lower educational attainment, reduced physical activity, higher obesity rates, and an increased burden of comorbidities, namely hyperlipidemia, hypertension, and diabetes mellitus. Notably, individuals with OAB exhibited significantly lower eGDR and elevated levels of systemic inflammatory biomarkers, including NLR, MLR, NMLR, SII, SIRI, AISI (all *P* < 0.05) (Table 1).

**Table 1:** Baseline characteristics of participants in the NHANES 2005–2018

Variables	Total (*n* = 31351)	Non-OAB (*n* = 24995)	OAB (*n* = 6356)	*P*
Age, years	47.00 (34.00, 60.00)	45.00 (32.00, 58.00)	59.00 (48.00, 71.00)	**< 0.001**
PIR	3.01 (1.50, 5.00)	3.18 (1.59, 5.00)	2.17 (1.16, 4.12)	**< 0.001**
Gender, n(%)				**< 0.001**
Male	15,734 (49.48)	13,087 (51.58)	2647 (38.25)	
Female	15,617 (50.52)	11,908 (48.42)	3709 (61.75)	
Ethnicity, n(%)				**< 0.001**
Mexican American	4937 (8.38)	3996 (8.56)	941 (7.41)	
Non-Hispanic White	13,607 (68.59)	11,033 (69.19)	2574 (65.33)	
Non-Hispanic Black	6499 (10.46)	4721 (9.38)	1778 (16.25)	
Other	6308 (12.58)	5245 (12.87)	1063 (11.01)	
Marital status, n(%)				**< 0.001**
Married or living with a partner	18,824 (64.02)	15,438 (64.96)	3386 (58.99)	
Widowed/divorced/separated	6922 (18.34)	4764 (16.27)	2158 (29.43)	
Never married	5605 (17.64)	4793 (18.77)	812 (11.59)	
Education, n(%)				**< 0.001**
Less than high school	7546 (15.36)	5380 (13.71)	2166 (24.23)	
High school or equivalent	7257 (23.43)	5728 (22.94)	1529 (26.05)	
College or above	16,548 (61.21)	13,887 (63.36)	2661 (49.72)	
Smoking status, n(%)				**< 0.001**
Never	17,141 (54.63)	14,044 (55.84)	3097 (48.12)	
Former	7698 (25.08)	5778 (24.07)	1920 (30.50)	
Now	6512 (20.29)	5173 (20.09)	1339 (21.37)	
Drinking status, n(%)				**< 0.001**
No	8845 (22.97)	6701 (21.77)	2144 (29.39)	
Yes	22,506 (77.03)	18,294 (78.23)	4212 (70.61)	
Physical activity, n(%)				**< 0.001**
Low	11,892 (33.40)	8741 (31.07)	3151 (45.86)	
High	19,459 (66.60)	16,254 (68.93)	3205 (54.14)	
BMI, n(%)				**< 0.001**
Underweight	474 (1.51)	391 (1.56)	83 (1.25)	
Normal weight	8431 (28.09)	7221 (29.69)	1210 (19.56)	
Overweight	10,479 (33.20)	8579 (33.75)	1900 (30.28)	
Obesity	11,967 (37.19)	8804 (35.00)	3163 (48.91)	
Hypertension, n(%)	13,486 (38.07)	9378 (34.06)	4108 (59.48)	**< 0.001**
Diabetes, n(%)	5928 (14.24)	3819 (11.71)	2109 (27.87)	**< 0.001**
Hyperlipidemia, n(%)	22,365 (70.32)	17,296 (68.56)	5069 (79.75)	**< 0.001**
eGDR^1^	8.67 (5.93, 10.15)	8.92 (6.37, 10.30)	6.39 (4.58, 9.00)	**< 0.001**
SII	473.35 (342.82, 660.47)	468.88 (342.11, 650.14)	504.00 (349.68, 713.00)	**< 0.001**
NLR	1.96 (1.50, 2.59)	1.95 (1.50, 2.55)	2.09 (1.54, 2.83)	**< 0.001**
AISI	252.31 (166.91, 388.80)	248.86 (166.19, 382.24)	272.91 (172.31, 424.41)	**< 0.001**
MLR	0.26 (0.21, 0.33)	0.26 (0.21, 0.33)	0.27 (0.21, 0.36)	**< 0.001**
NMLR	2.23 (1.74, 2.90)	2.21 (1.73, 2.86)	2.36 (1.77, 3.18)	**< 0.001**
SIRI	1.05 (0.73, 1.52)	1.04 (0.72, 1.49)	1.14 (0.76, 1.70)	**< 0.001**

Bold values indicate the statistically significant *P*-values

Median (IQR) for continuous variables: *p* for weighted Kruskal-Wallis test. % for categorical variables: *p* for weighted chi-square test. OAB, overactive bladder; PIR, poverty-to-income ratio; BMI, body mass index; eGDR, estimated glucose disposal rate; NLR, neutrophil-to-lymphocyte ratio; MLR, monocyte-to-lymphocyte ratio; NMLR, neutrophil-monocyte-to-lymphocyte ratio; SII, systemic immune-inflammation index; SIRI, systemic inflammation response index; AISI, aggregate index of systemic inflammation

¹eGDR is expressed in mg/kg/min, approximating the units used in hyperinsulinemic-euglycemic clamp studies for comparability

### Association between eGDR and OAB

In both univariate and multivariable analyses, eGDR was significantly inversely associated with OAB (Table 2). In the unadjusted model, eGDR demonstrated a protective effect: each unit increase corresponded to a 20% reduction in OAB odds (OR = 0.80; 95% CI: 0.79–0.81; *P* < 0.001). This inverse relationship remained robust after controlling for confounding variables; the association was attenuated but still significant in both the partially adjusted Model 2 (OR = 0.86; 95% CI: 0.85–0.88; *P* < 0.001) and the fully adjusted Model 3 (OR = 0.89; 95% CI: 0.87–0.91; *P* < 0.001), indicating a 14% and 11% reduction in OAB odds, respectively. To further characterize the relationship between eGDR and OAB risk, we categorized eGDR into quartiles (Q1–Q4). In Model 3, compared with Q1, higher eGDR quartiles were independently correlated with reduced OAB risk: Q2 (OR = 0.66; 95% CI: 0.60–0.74; *P* < 0.001), Q3 (OR = 0.59; 95% CI: 0.52–0.68; *P* < 0.001), and Q4 (OR = 0.52; 95% CI: 0.42–0.64; *P* < 0.001), demonstrating a clear dose-dependent decrease in OAB risk across increasing eGDR quartiles.

**Table 2:** Multivariate logistic regression analysis of eGDR with OAB

Variables	Model 1		Model 2		Model 3	
	OR (95%CI)	*P*	OR (95%CI)	*P*	OR (95%CI)	*P*
eGDR	0.80 (0.79 ~ 0.81)	**< 0.001**	0.86 (0.85 ~ 0.88)	**< 0.001**	0.89 (0.87 ~ 0.91)	**< 0.001**
1	1.00 (Reference)		1.00 (Reference)		1.00 (Reference)	
2	0.52 (0.47 ~ 0.57)	**< 0.001**	0.61 (0.55 ~ 0.67)	**< 0.001**	0.66 (0.60 ~ 0.74)	**< 0.001**
3	0.32 (0.29 ~ 0.35)	**< 0.001**	0.48 (0.43 ~ 0.54)	**< 0.001**	0.59 (0.52 ~ 0.68)	**< 0.001**
4	0.21 (0.18 ~ 0.24)	**< 0.001**	0.37 (0.32 ~ 0.44)	**< 0.001**	0.52 (0.42 ~ 0.64)	**< 0.001**
*P* for trend	**< 0.001**		**< 0.001**		**< 0.001**	

Bold values indicate the statistically significant *P*-values

eGDR, estimated glucose disposal rate; OAB, overactive bladder; OR, odds ratio; CI, confidence interval; Model 1: unadjusted; Model 2: adjusted for age, gender and ethnicity; Model 3: adjusting for variables in Model 2 and with additional adjustment for marital status, education level, poverty-to-income ratio, smoking status, drinking status, physical activity, body mass index, and hyperlipidemia

**Fig. 2:**
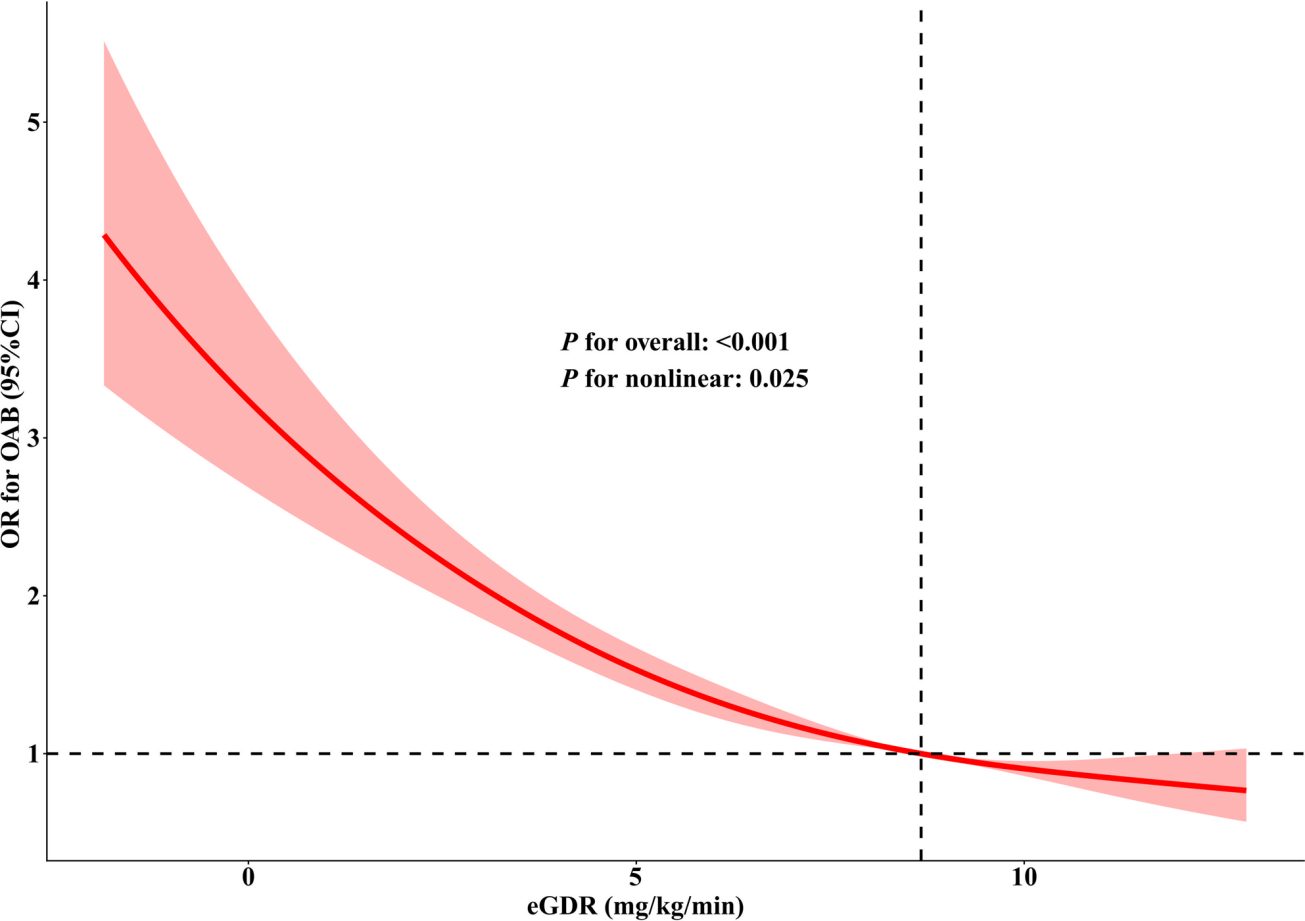
The associations between eGDR and the risk of OAB. Dose-response relationships were assessed using RCS models with knots placed at the 15th, 35th, 65th, and 90th percentiles of eGDR. The model was adjusted for age, gender, ethnicity, marital status, education level, poverty-to-income ratio, smoking status, drinking status, physical activity, body mass index, and hyperlipidemia. eGDR, estimated glucose disposal rate; OAB, overactive bladder; OR, odds ratio; CI, confidence interval

A non-linear negative association between eGDR and OAB risk was visualized using RCS regression (Fig. 2). After controlling for confounding factors in Model 3, this relationship proved robust and maintained its statistical significance (overall *P* < 0.001; nonlinear *P* = 0.025). Sensitivity analysis using unimputed data confirmed stable association between eGDR and OAB. Detailed results are presented in Table S3. Moreover, we examined the associations between TyG and HOMA-IR with OAB in the imputed dataset as a sensitivity analysis. In unadjusted and partially adjusted models, higher HOMA-IR and TyG were significantly associated with increased OAB risk; however, these associations attenuated and became non-significant after full adjustment for all covariates. This suggests that eGDR may be a more reliable indicator of OAB risk than HOMA-IR or TyG, particularly when accounting for comprehensive confounding. Detailed results are presented in Tables S4-S5.

### Subgroup analyses

**Fig. 3:**
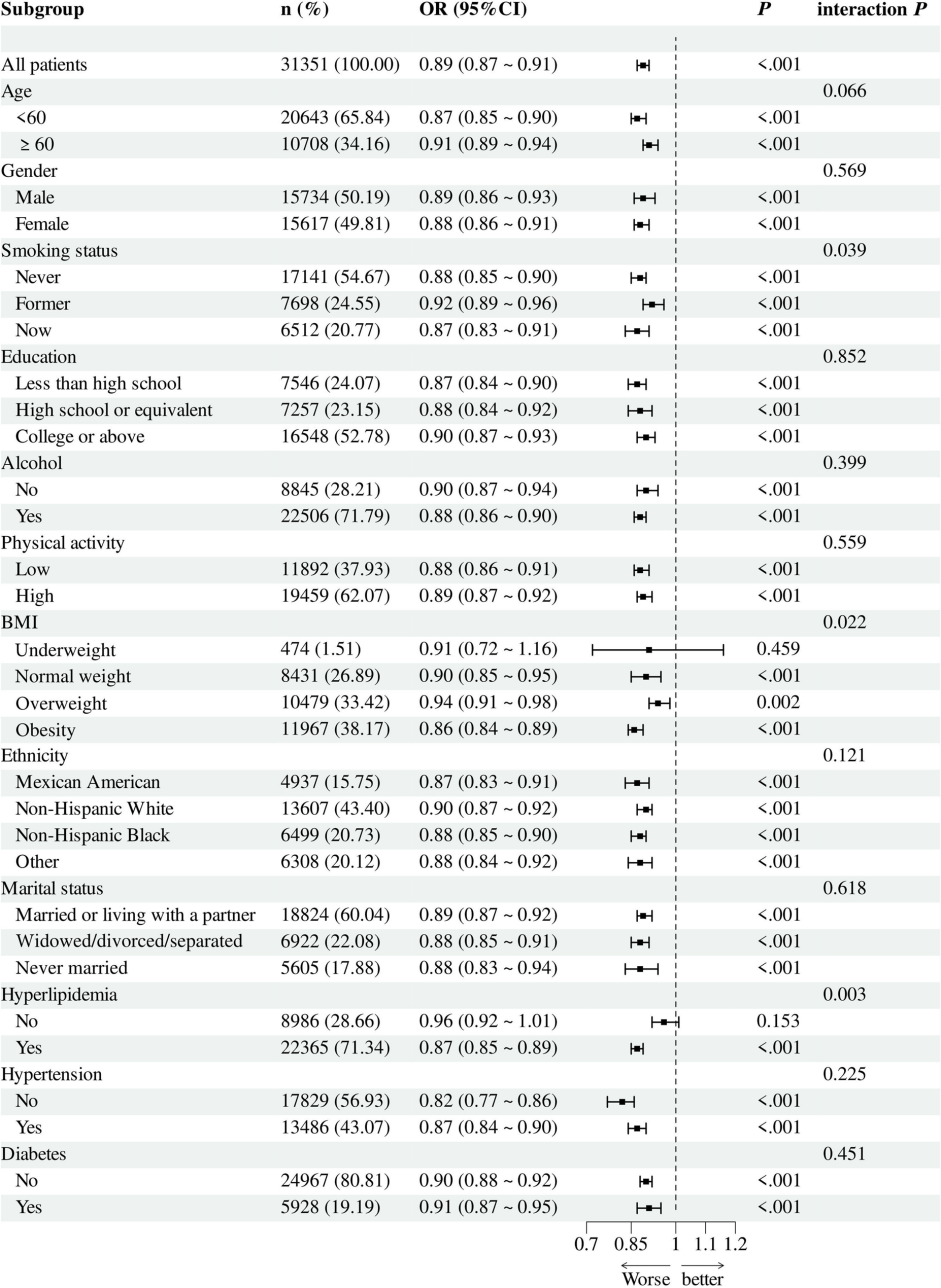
The associations between eGDR and the risk of OAB in various subgroups. OR, odds ratio; CI, confidence interval; BMI, body mass index

Subgroup analyses revealed significant variations in the eGDR-OAB correlation based on smoking status, BMI, and hyperlipidemia (interaction *P* < 0.05), indicating possible effect modification by these factors. Notably, the inverse association did not reach statistical significance among underweight individuals or those without hyperlipidemia (*P* > 0.05). In contrast, no statistically significant interaction effects were detected across other subgroups, including age, gender, ethnicity, drinking status, physical activity, education level, marital status, hypertension, and diabetes mellitus (all interaction *P* > 0.05) (Fig. 3).

### Mediation effects of inflammation

**Fig. 4:**
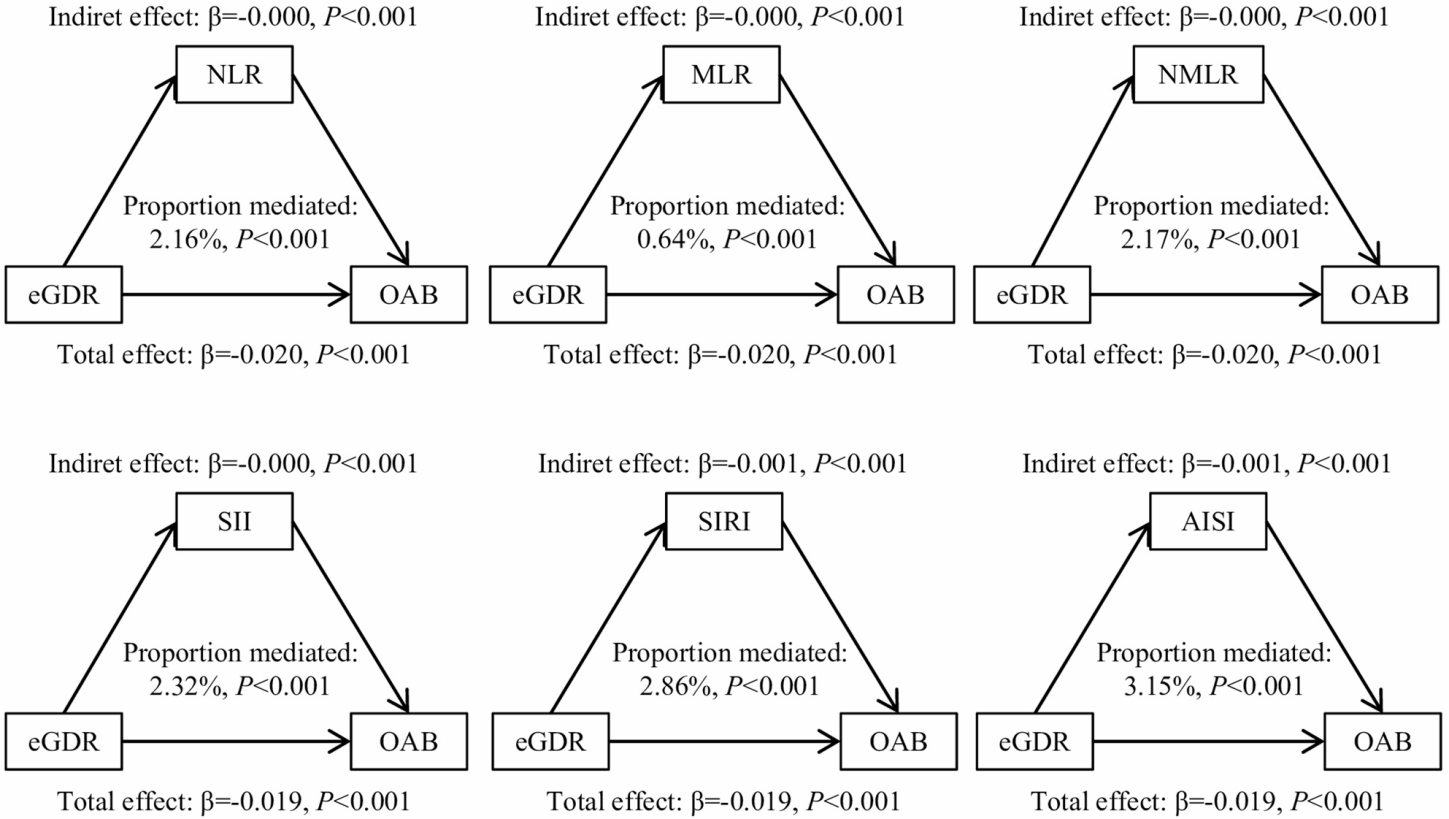
The mediation effects of systemic inflammatory biomarkers in the associations between eGDR and OAB.Proportions mediated were small (< 3.2% for all biomarkers), suggesting that the majority of the eGDR–OAB association operates through pathways other than systemic inflammation. The model was adjusted for age, gender, ethnicity, marital status, education level, poverty-to-income ratio, smoking status, drinking status, physical activity, body mass index, and hyperlipidemia. eGDR, estimated glucose disposal rate; OAB, overactive bladder; NLR, neutrophil-to-lymphocyte ratio; MLR, monocyte-to-lymphocyte ratio; NMLR, neutrophil-monocyte-to-lymphocyte ratio; SII, systemic immune-inflammation index; SIRI, systemic inflammation response index; AISI, aggregate index of systemic inflammation

Participants with OAB showed markedly elevated levels of systemic inflammatory biomarkers (NLR, MLR, NMLR, SII, SIRI, AISI) compared to those without OAB (Table 1). To investigate whether these systemic inflammatory biomarkers mediate the link between eGDR and OAB risk, we performed mediation analyses (Fig. 4). The results indicated that NLR, MLR, NMLR, SII, SIRI, and AISI accounted for 2.16%, 0.64%, 2.17%, 2.32%, 2.86%, and 3.15% of the total effect, respectively. AISI showed the highest mediation proportion (3.15%), but this effect remains modest.

## Discussion

Using data from the nationally representative NHANES, our study employed a cross-sectional design to examine the interplay between eGDR, systemic inflammation, and OAB risk. To our knowledge, this is the first study to systematically evaluate inflammatory mediation in the eGDR-OAB relationship. Clinically, our findings suggest that patients with low eGDR indicating advanced IR may be at substantially higher risk for OAB. The presence of a dose-dependent and nonlinear relationship enhances the biological credibility of this association. Additionally, multiple inflammatory markers, including NLR, MLR, SII, SIRI, and AISI, partially mediated the eGDR-OAB association. However, the mediation proportions remained modest (0.64–3.15%), indicating that inflammation may have contributed to the observed association to some extent, but that the majority of the association is likely explained by non-inflammatory mechanisms.

Given the impracticality of the clamp technique in real-world settings, surrogate IR markers are essential. HOMA-IR is well-validated, but its dependence on insulin measurement limits scalability [9]. The TyG index addresses this gap, and accumulating evidence supports its superior performance over HOMA-IR [23]. eGDR integrates HbA1c, waist circumference, and hypertension status into a composite marker reflecting multifaceted metabolic dysfunction, which correlates highly with clamp-derived insulin sensitivity, and offers an even more comprehensive, clinically grounded alternative [24]. By comparing HOMA-IR, TyG, and eGDR within a single representative cohort, we found that only eGDR retained a statistically significant association with OAB risk after full adjustment for all covariates, which suggests that eGDR may serve as a more robust biomarker for identifying individuals at elevated risk of OAB. eGDR has been validated for cardiovascular risk prediction [11, 25, 26], and extending its application to OAB provides insights into the metabolic-urological continuum.

The development of OAB involves multiple interrelated pathophysiological pathways. These are broadly classified into myogenic, neurogenic, and urotheliogenic mechanisms, alongside other contributing factors such as metabolic syndrome, mood disorders, sex hormone imbalances, changes in the urinary microbiome, gastrointestinal issues, and dysfunctions of the autonomic nervous system [27]. As a core pathogenic element in obesity, type 2 diabetes, and metabolic syndrome, IR is postulated to elevate OAB risk through mechanisms such as promoting chronic pelvic ischemia, compromising uroepithelial function, and dysregulating the autonomic nervous system [7, 27, 28]. At the molecular level, experimental studies demonstrate that insulin promotes detrusor relaxation in both human and rodent bladders by stimulating the PI3K/AKT/eNOS cascade in urothelial cells; and obesity-associated endoplasmic reticulum stress triggers localized IR within the bladder wall, which is a pivotal event in the pathogenesis of OAB [29]. Tadalafil improves bladder overactivity by restoring insulin-induced detrusor relaxation via this same pathway in rats [30]. Furthermore, emerging evidence suggests that dysregulation of insulin signaling in the bladder urothelium may represent a common mechanistic link underlying OAB in rat models of metabolic syndrome programmed by maternal and post-weaning fructose exposure [31]. Our findings, which reveal an inverse relationship between eGDR and OAB risk, are biologically grounded and find support in prior mechanistic studies. Notably, HOMA-IR was markedly higher among women with OAB than in healthy controls [6]. Other studies have also reported inverse associations between the TyG-related indices and OAB risk [12, 13]. Our findings corroborate the majority of existing evidence. Furthermore, we employed eGDR, a validated composite indicator of insulin sensitivity, and confirmed its association with OAB in a large and representative sample. The stepwise decrease in OAB risk with increasing eGDR quartiles supports a potential dose-response relationship. The nonlinear trend shown by RCS curves implies that the effect of eGDR on OAB may not be linear; instead, it may follow a threshold model, where risk increases sharply at lower eGDR levels but plateaus at higher eGDR. This has important implications for precision prevention strategies. Although the P-value for the nonlinearity test was statistically significant (*P* = 0.025), it approached the nominal threshold of 0.05, and the RCS curve appears largely monotonic—suggesting that the evidence for nonlinearity is modest. Thus, we interpret this finding as suggestive rather than conclusive, and emphasize that clinical inference should prioritize the overall dose–response pattern over marginal statistical significance. Subgroup analyses revealed statistically significant interactions in populations stratified by smoking, BMI, and hyperlipidemia. These factors may independently contribute to systemic inflammation and interact with eGDR to elevate OAB susceptibility. The eGDR–OAB association appeared stronger in obese individuals, possibly reflecting compounded metabolic burden. Given that obesity is a major contributor to IR, its coexistence with low eGDR may synergistically heighten OAB risk through inflammatory and metabolic mechanisms. Notably, this association remained consistent across most subgroups, enhancing the generalizability and reliability of our findings. Inflammatory processes play a pivotal role in normal physiological functions and the pathogenesis of diseases affecting various organ systems [32]. Prior research indicates that inflammation may function as an intermediary factor connecting diabetes mellitus to the onset of OAB [33]. Additional evidence also supports the interplay between metabolic disturbances, inflammation, and lower urinary tract dysfunction [34]. Our study found that the indirect effects of inflammatory markers on the eGDR-OAB association were statistically significant, with the AISI showing the highest mediation proportion at 3.15%. The state of IR is characterized by enhanced macrophage recruitment to adipose tissue and excessive secretion of pro-inflammatory cytokines like TNF-α and IL-6, thereby fostering a state of persistent systemic inflammation [35, 36]. Chronic low-grade inflammation appears to be an integral component of OAB pathogenesis. Through multiple interconnected pathways, including neurogenic, myogenic, and urothelial mechanisms, inflammatory mediators can directly damage or sensitize bladder afferent nerves, alter detrusor excitability and contractility, and compromise urothelial barrier integrity, ultimately manifesting as OAB symptoms such as urinary urgency and increased voiding frequency [27, 37, 38].

While our findings suggest eGDR is associated with OAB risk, clinical implementation as a screening tool requires: (1) prospective validation of predictive performance, (2) establishment of optimal cut-off values, and (3) comparative studies demonstrating advantages over existing IR indices. At present, our study supports eGDR as a research tool for understanding metabolic contributions to OAB pathophysiology, but further work is needed before clinical application. In this study, although the mediation proportion was relatively low, at only about 0.64–3.15%, it is commonly observed in large-scale population studies and carries significant biological relevance indicating that inflammation represents a key, but not exclusive pathway. The mediating effect of inflammation represents a specific and measurable pathophysiological link between eGDR and OAB, providing a clear direction for future mechanistic investigations and targeted interventions.

This study, based on the NHANES database, is the first to systematically evaluate the mediating roles of multiple systemic inflammatory biomarkers in the association between eGDR and OAB. Utilizing a population-based, nationally representative sample ensures broad external validity for our conclusions. We employed multivariable adjustment models, dose-response analyses, comprehensive subgroup stratification, and formal mediation analysis to rigorously evaluate the robustness of our results. Both eGDR and the inflammatory markers are derived from routine clinical laboratory tests, facilitating potential translation into clinical practice. However, it is important to note the limitations of this study. First, the cross-sectional design limits our ability to draw definitive causal conclusions regarding the interplay between eGDR, inflammation, and OAB. Reverse causality remains possible for example, OAB might reduce physical activity or promote weight gain, potentially worsening IR. Second, we did not evaluate diagnostic performance of eGDR for OAB, including sensitivity, specificity, and area under the receiver operating characteristic curve (AUC), thereby limiting conclusions regarding its utility as a screening tool. At present, eGDR should be interpreted primarily as an epidemiological marker rather than a clinically validated risk stratification tool. Third, the reliance on self-reported data for OAB diagnosis carries inherent limitations, as the subjective nature of symptoms may introduce both recall and classification bias. Fourth, although extensive adjustments have been made for known confounding factors, there may still be unmeasured confounding factors, such as dietary patterns or specific medication histories. Finally, our mediation analysis suggests inflammation may statistically explain a small portion of the eGDR-OAB relationship. However, the cross-sectional nature of NHANES precludes causal conclusions [39]. Prospective studies with repeated measures of eGDR, inflammation, and OAB symptoms, along with experimental validation, are needed to confirm inflammatory pathways as causal mediators. Ultimately, these efforts may inform novel strategies targeting improved insulin sensitivity and anti-inflammatory interventions for the prevention and management of OAB.

## Conclusion

Our findings provide the first evidence of a nonlinear inverse relationship connecting eGDR to OAB risk, with systemic inflammatory biomarkers partially mediating this relationship, although this effect remains modest. The eGDR may serve as a marker for OAB risk stratification in future prospective studies. The mediating effect of inflammation provides a quantifiable pathophysiological pathway linking eGDR and OAB, supporting the development of targeted interventions. Future research should employ prospective cohort designs to enable robust causal inference and in-depth mechanistic exploration.

## Electronic Supplementary Material

Below is the link to the electronic supplementary material.

Supplementary material 1.

## Data Availability

The data supporting the findings of this study are publicly available through the NHANES at https://www.cdc.gov/nchs/nhanes/.
