# Supplementary material for: Associations of estimated glucose disposal rate and overactive bladder and the potential mediating role of systemic inflammation: NHANES 2005–2018
Source: Diabetol Metab Syndr. 2026 May 19;18:154. doi: 10.1186/s13098-026-02182-4 (PMC13361660; doi:10.1186/s13098-026-02182-4)
Supplement: Supplementary file 1 — Supplementary material 1. [file 13098_2026_2182_MOESM1_ESM.docx]

**Supplementary Material**

**Table S1.** Criteria for conversion of symptom frequencies recorded in NHANES and OABSS scores.

| According to NHANES score | According to OABSS score |
| --- | --- |
| **Urge urinary incontinence frequency** | **Urge urinary incontinence score** |
| Never | 0 |
| Less than once a month | 1 |
| A few times a month | 1 |
| A few times a week | 2 |
| Every day or night | 3 |
| **Nocturia frequency score** | **Nocturia score** |
| 0 | 0 |
| 1 | 1 |
| 2 | 2 |
| 3 | 3 |
| 4 | 3 |
| 5 or more | 3 |

NHANES, National Health and Nutrition Examination Survey; OABSS, overactive bladder symptom score.

**Table S2.** The calculation methods for inflammation biomarkers.

| Variable | Calculation formula |
| --- | --- |
| NLR | Neutrophil counts / lymphocyte counts |
| MLR | Monocyte counts / lymphocyte counts |
| NMLR | (monocyte counts + neutrophil counts) / lymphocyte counts |
| SIRI | Neutrophil counts × monocyte counts / lymphocyte counts |
| SII | Platelet counts × neutrophil counts / lymphocyte counts |
| AISI | Platelet counts × neutrophil counts × monocyte counts / lymphocyte counts |

NLR, neutrophil-to-lymphocyte ratio; MLR, monocyte-to-lymphocyte ratio; NMLR, neutrophil-monocyte-to-lymphocyte ratio; SII, systemic immune-inflammation index; SIRI, systemic inflammation response index; AISI, aggregate index of systemic inflammation.

**Table S3.** Multivariate logistic regression analysis of the association between eGDR and OAB using unimputed data.

| Variables | Model 1 | |  | Model 2 | |  | Model 3 | |
| --- | --- | --- | --- | --- | --- | --- | --- | --- |
|  | OR (95%CI) | *P* |  | OR (95%CI) | *P* |  | OR (95%CI) | *P* |
| eGDR | 0.80 (0.79 ~ 0.81) | **<0.001** |  | 0.86 (0.85 ~ 0.88) | **<0.001** |  | 0.90 (0.88 ~ 0.92) | **<0.001** |
| eGDR quartile | | | | | | | | |
| 1 | 1.00 (Reference) |  |  | 1.00 (Reference) |  |  | 1.00 (Reference) |  |
| 2 | 0.52 (0.47 ~ 0.57) | **<0.001** |  | 0.61 (0.55 ~ 0.67) | **<0.001** |  | 0.71 (0.63 ~ 0.80) | **<0.001** |
| 3 | 0.32 (0.29 ~ 0.35) | **<0.001** |  | 0.48 (0.43 ~ 0.54) | **<0.001** |  | 0.61 (0.52 ~ 0.71) | **<0.001** |
| 4 | 0.21 (0.18 ~ 0.24) | **<0.001** |  | 0.37 (0.32 ~ 0.44) | **<0.001** |  | 0.58 (0.45 ~ 0.75) | **<0.001** |
| *P* for trend | **<0.001** | |  | **<0.001** | |  | **<0.001** | |

**Notes:** eGDR, estimated glucose disposal rate; OAB, overactive bladder; OR, odds ratio; CI, confidence interval; Model 1: unadjusted; Model 2: adjusted for age, gender and ethnicity; Model 3: adjusting for variables in Model 2 and with additional adjustment for marital status, education level, poverty-to-income ratio, smoking status, drinking status, physical activity, body mass index, and hyperlipidemia.

### **Table S4.** Multivariate logistic regression analysis of the association between HOMA-IR and OAB.

| Variables | Model 1 | |  | Model 2 | |  | Model 3 | |
| --- | --- | --- | --- | --- | --- | --- | --- | --- |
|  | OR (95%CI) | *P* |  | OR (95%CI) | *P* |  | OR (95%CI) | *P* |
| HOMA-IR | 1.02 (1.01 ~ 1.03) | **<0.001** |  | 1.02 (1.01 ~ 1.02) | **<0.001** |  | 1.00 (1.00 ~ 1.01) | **<0.424** |
| HOMA-IR quartile | | | | | | | | |
| 1 | 1.00 (Reference) |  |  | 1.00 (Reference) |  |  | 1.00 (Reference) |  |
| 2 | 1.30 (1.09 ~ 1.55) | **0.004** |  | 1.19 (1.00 ~ 1.42) | **0.050** |  | 0.99 (0.82 ~ 1.19) | **0.906** |
| 3 | 1.45 (1.22 ~ 1.71) | **<0.001** |  | 1.26 (1.05 ~ 1.50) | **0.012** |  | 0.95 (0.77 ~ 1.18) | **0.662** |
| 4 | 1.99 (1.66 ~ 2.39) | **<0.001** |  | 1.72 (1.43 ~ 2.06) | **<0.001** |  | 1.07 (0.85 ~ 1.34) | **0.563** |
| *P* for trend | **<0.001** | |  | **<0.001** | |  | **0.380** | |

**Notes:** HOMA-IR, Homeostatic Model Assessment of Insulin Resistance; OAB, overactive bladder; OR, odds ratio; CI, confidence interval; Model 1: unadjusted; Model 2: adjusted for age, gender and ethnicity; Model 3: adjusting for variables in Model 2 and with additional adjustment for marital status, education level, poverty-to-income ratio, smoking status, drinking status, physical activity, body mass index, and hyperlipidemia.

### **Table S5.** Multivariate logistic regression analysis of the association between TyG and OAB.

| Variables | Model 1 | |  | Model 2 | |  | Model 3 | |
| --- | --- | --- | --- | --- | --- | --- | --- | --- |
|  | OR (95%CI) | *P* |  | OR (95%CI) | *P* |  | OR (95%CI) | *P* |
| TyG | 1.41 (1.31 ~ 1.51) | **<0.001** |  | 1.31 (1.21 ~ 1.43) | **<0.001** |  | 1.06 (0.95 ~ 1.17) | **0.312** |
| TyG quartile | | | | | | | | |
| 1 | 1.00 (Reference) |  |  | 1.00 (Reference) |  |  | 1.00 (Reference) |  |
| 2 | 1.33 (1.14~ 1.56) | **<0.001** |  | 1.16 (0.98 ~ 1.38) | **0.093** |  | 0.96 (0.80 ~ 1.15) | **0.653** |
| 3 | 1.59 (1.34 ~ 1.89) | **<0.001** |  | 1.33 (1.11 ~ 1.61) | **0.003** |  | 0.98 (0.80 ~ 1.21) | **0.873** |
| 4 | 1.90 (1.63 ~ 2.22) | **<0.001** |  | 1.57 (1.32 ~ 1.87) | **<0.001** |  | 1.01 (0.82 ~ 1.25) | **0.914** |
| *P* for trend | **<0.001** | |  | **<0.001** | |  | **0.782** | |

**Notes:** TyG, triglyceride-glucose index; OAB, overactive bladder; OR, odds ratio; CI, confidence interval; Model 1: unadjusted; Model 2: adjusted for age, gender and ethnicity; Model 3: adjusting for variables in Model 2 and with additional adjustment for marital status, education level, poverty-to-income ratio, smoking status, drinking status, physical activity, body mass index, and hyperlipidemia.
